# Control of parallelized bioreactors II: probabilistic quantification of carboxylic acid reductase activity for bioprocess optimization

**DOI:** 10.1007/s00449-022-02797-7

**Published:** 2022-10-28

**Authors:** Nikolas von den Eichen, Michael Osthege, Michaela Dölle, Lukas Bromig, Wolfgang Wiechert, Marco Oldiges, Dirk Weuster-Botz

**Affiliations:** 1 Chair of Biochemical Engineering, Technical University of Munich, Garching, Germany; 2 Institute of Biotechnology: IBG-1, Forschungszentrum Jülich GmbH, Jülich, Germany; 3 Institute of Biotechnology, RWTH Aachen University, Aachen, Germany; 4 Computational Systems Biotechnology (AVT.CSB), RWTH Aachen University, Aachen, Germany

**Keywords:** Automation, Bioprocess development, Bayesian modelling, *Escherichia coli*, Protein expression, Stirred-tank bioreactors, Whole-cell catalysis

## Abstract

Autonomously operated parallelized mL-scale bioreactors are considered the key to reduce bioprocess development cost and time. However, their application is often limited to products with very simple analytics. In this study, we investigated enhanced protein expression conditions of a carboxyl reductase from *Nocardia otitidiscaviarum* in *E. coli*. Cells were produced with exponential feeding in a L-scale bioreactor. After the desired cell density for protein expression was reached, the cells were automatically transferred to 48 mL-scale bioreactors operated by a liquid handling station where protein expression studies were conducted. During protein expression, the feed rate and the inducer concentration was varied. At the end of the protein expression phase, the enzymatic activity was estimated by performing automated whole-cell biotransformations in a deep-well-plate. The results were analyzed with hierarchical Bayesian modelling methods to account for the biomass growth during the biotransformation, biomass interference on the subsequent product assay, and to predict absolute and specific enzyme activities at optimal expression conditions. Lower feed rates seemed to be beneficial for high specific and absolute activities. At the optimal investigated expression conditions an activity of $1153\ U\ mL^{-1}$ was estimated with a $90\%$ credible interval of $[992, 1321]\ U\ mL^{-1}$. This is about 40-fold higher than the highest published data for the enzyme under investigation. With the proposed setup, 192 protein expression conditions were studied during four experimental runs with minimal manual workload, showing the reliability and potential of automated and digitalized bioreactor systems.

## Introduction

Because of the need to perform time-consuming and labor-intensive experiments for bioprocess development, miniaturized and automated bioreactor systems have been developed with which a variety of process parameters can be screened rapidly [1, 2]. Parallel microbioreactor systems are often coupled with a pipetting robot (liquid handling station, LHS) to use the flexibility of the LHS for at-line process analysis [3–5]. It has been shown that microbioreactor systems can yield scalable results for both biomass growth and product formation [4, 6, 7]. Heterologous proteins are usually over expressed by cloning the encoding gene downstream from a regulated promoter in a suitable host to allow for cheap and simple protein production [8]. To reduce adverse effects on biomass growth due to the formation of the heterologous protein, the cell formation phase is usually separated from the product formation phase [6, 9–11]. This is accomplished by making protein formation controllable by inducers and activating it only after the desired cell density has been reached [11].

To be able to study these phases separately, we developed a fully automated system with which cells are produced firstly in the L-scale stirred-tank reactor and are transferred secondly to parallel operated mL-scale stirred-tank reactors after reaching the desired cell density for protein expression [12]. Protein expression studies and product analyses are then conducted at the mL-scale. In the past, however, both this system and other microbioreactor systems have predominantly investigated model proteins with highly simplified product analytics [12–14] or product analysis involved manual processing steps [3]. Manual steps in the context of automated process development carry the risk of merely shifting the effort required for bioprocess development rather than reducing it. Therefore, the goal of this study was to apply a fully automated parallel bioreactor system for studies on the expression of a carboxyl reductase (CAR) in *Escherichia coli* (*E. coli*). Carboxyl reductases are a class of large enzymes (approximately 130 kDa) used for the selective reduction of aldehydes from carboxylic acids in various applications [15]. Chemicals resulting from those reactions can be used in the manufacturing of drugs for cardiovascular, antiparasitic, and anticholinergic applications [15–17]. To quantify the expression success in *E. coli*, whole-cell biotransformations were performed in deep-well-plates (DWP) for the determination of enzyme activity [18]. To keep the necessary robotic equipment as simple as possible, the analysis of the biotransformation was carried out without prior separation of the cells. However, this necessitated the model-based evaluation of the enzyme activity, since the photometric detection of the biotransformation product is disturbed by the growing cells.

### Aim of this study

To demonstrate the potential of miniaturized bioprocess development, a total of 192 protein expressions and 264 whole-cell biotransformations were performed in 4 sequential experiments. The feed rate during protein expression and the inducer concentration were examined in a total of 42 different combinations. These two parameters were selected because they have been shown in the past to be critical for heterologous protein production [11, 12].

A detailed computational model of the experimental process was implemented to describe observed absorbance from underlying biomass and product concentrations. The model captured not only the concentrations at observed time points, but also comprises mechanistic descriptions of how these concentrations result from otherwise unobservable parameters and key performance indicators (KPIs) such as specific enzyme activity. Using Markov-chain Monte Carlo (MCMC) methods, we quantified the posterior probability distributions of model parameters and variables, thereby obtaining uncertainty estimates for KPIs of interest.

Through Bayesian modeling, we determined the biomass-specific and absolute enzyme activity within the investigated parameter space and predicted optimal expression conditions for the CAR protein in the *E. coli* process.

## Materials and methods

### Bacterial strain

*E. coli* K12 MG1655 RARE (#61440 at Addgene, Watertown, USA) with a pETDuet plasmid with a carboxylase gene from *Nocardia otitidiscaviarum* and a pyrophosphatase from *E. coli* (EcPPase) under the control of a T7 promoter [15] kindly provided by Prof. Dörte Rother (Forschungszentrum Jülich, Jülich, Germany) was used for all cultivations. The recombinant *E. coli* cells were stored as cryo-cultures at -80 $^\circ$C after mixing the cell suspension 1:1 with a 50 % (v/v) glycerol solution.

### Media

Seed cultures were grown at 37 $^\circ$C with LB-medium ($5\ g\ L^{-1}$ yeast extract, $10\ g\ L^{-1}$ peptone, $5\ g\ L^{-1}$ NaCl, $50\ mg\ L^{-1}$ ampicillin, pH 7.5) in 1 L shake flasks with baffles at 150 rpm with a working volume of 100 mL. The pH of the LB-medium was adjusted with 2 M NaOH prior to autoclaving (20 min at 121 $^\circ$C). Sterile-filtered ampicillin was added aseptically after autoclaving the LB-medium.

All cultivations on the mL- and L-scales were carried out with a defined minimal medium [19]. The final concentrations in the medium were as follows: $8.4\ mg\ L^{-1}$ ethylenediaminetetraacetic acid (EDTA), $8.4 mg\ L^{-1}$ $CoCl_{2}*6H_{2}O$, $15\ mg\ L^{-1} MnCl_{2}*4H_{2}O$, $1.5\ mg\ L^{-1} CuCl_{2}*2H_{2}O$, $3\ mg\ L^{-1} H_{3}BO_{3}$, $2.5\ mg\ L^{-1} Na_{2}MoO_{4}*2H_{2}O$, $13\ mg\ L^{-1} Zn(CH_{3}COO)_{2}*2H_{2}O$, $100\ mg\ L^{-1}$ Fe(III)citrate, $13.3\ g\ L^{-1} KH_{2}PO_{4}$, $4\ g\ L^{-1} (NH_{4})_{2}HPO_{4}$, $1.7\ g\ L^{-1}\ \mathrm {citric\ acid}*H_{2}O$, $2.4\ g\ L^{-1}$ NaOH, $1.2\ g\ L^{-1} MgSO_{4}*7H_{2}O$, $50\ mg\ L^{-1}$ ampicillin. The pH was not adjusted prior to addition to the bioreactor. The initial glucose concentration was $5\ g\ L^{-1}$. The feed medium consisted of $500\ g\ L^{-1}$ glucose with $12.5\ g\ L^{-1} MgSO_{4}$ in fed-batch processes on the L-scale. For the mL-scale, the feed medium varied depending on the applied feed rate. For the experiments with a feed rate of $4.8\ g\ L^{-1} h^{-1}$ the feed medium consisted of $300\ g\ L^{-1}$ glucose with $7.5\ g\ L^{-1} MgSO_{4}$. For the experiment with the feed rates varied from 2-4 $g\ L^{-1}h^{-1}$ the feed medium consisted of $200\ g\ L^{-1}$ glucose with $5\ g\ L^{-1} MgSO_{4}$. For the experiment with the feed rates varied from 1 - $2\ g\ L^{-1} h^{-1}$ the feed medium consisted of $100\ g\ L ^{-1}$ glucose with $2.5\ g\ L^{-1} MgSO_{4}$. The varying feed concentrations were necessary to allow different feed rates with the same feed dosage frequency by the liquid handling system (LHS) while maintaining comparable reactor volumes. Ignoring the uneven effect of evaporation, the glucose concentration is not proportional to the feed rate.

Prior to transfer of the cells from the L-scale to mL-scale, 0.5 % (v/v) antifoam agent (Antifoam 204, Sigma-Aldrich / Merck KGaA, Darmstadt, Germany) was added aseptically. $MgSO_{4}*7H_{2}O$, glucose and ampicillin were added aseptically after autoclaving of the medium. $MgSO_{4}*7H_{2}O$ and glucose were autoclaved separately, ampicillin was sterile-filtered.

### Seed culture

Seed culture preparation was performed in 1000 mL baffled shake flasks inoculated with 500 $\upmu$L of the cryo-culture in 100 mL LB medium. The cells were grown for 7.5 h in a rotary shaker (Multitron, Infors, Bottmingen-Basel, Switzerland) at 150 rpm and 37 $^\circ$C.

### Stirred-tank bioreactors

The cultivation procedure was adapted from von den Eichen et al. [12]. A parallel bioreactor system on an L-scale (DASGIP Parallel Bioreactor System, Eppendorf AG, Hamburg, Germany) with a working volume of 0.5 L was used for a cultivation consisting of a batch (initial glucose concentration $5\ g\ L^{-1}$) and subsequent fed-batch phase with $\mu _{set} = 0.1\ h^{-1}$ to produce a sufficient cell density for the induction of the protein production. The bioreactor was equipped with a DO probe (Visiferm DO ECS 225 H0, Hamilton Bonaduz AG, Bonaduz, Switzerland). The fed-batch phase was started automatically based on the slow decline of the dissolved oxygen (DO) signal followed by a steep rise above 75 % during the batch phase. The pH was controlled at pH 7.0 with a pH probe (EasyFerm Plus PHI K8 225, Hamilton Bonaduz AG, Bonaduz, Switzerland). During the cultivation on a L-scale, the temperature was 37 $^\circ$C. The exponential feeding was stopped after 23 h process time at a cell density $> 10\ g\ L^{-1}$ to make sure that the final dry cell mass concentration in the subsequently used stirred-tank bioreactors will not exceed $40\ g\ L^{-1}$ to avoid any disturbance of the fluorometric pH sensors [20].

After 23 h process time the cell broth from the L-scale bioreactor was automatically transferred to a bioreaction unit with 48 mL-scale stirred-tank-bioreactors operated with gas-inducing stirrers (bioREACTOR48, 2mag AG, Munich, Germany). The transfer procedure has been described in von den Eichen et al. [12]. Due to more time-efficient pump control compared to our previous publication, the total time needed for the transfer was reduced to approximately 25 minutes. Sterile single-use bioreactors with a working volume of 10 mL with baffles (HTBD, 2mag AG, Munich, Germany) with fluorometric sensors for online DO and pH measurements were used for all experiments (PSt3-HG sensor for DO, LG1 sensor for pH, PreSens GmbH, Regensburg, Germany). During cultivations on an mL-scale, the temperature was lowered to 30 $^\circ$C. The bioreaction unit was placed on the working table of a liquid handling system (LHS, Microlab STARlet, Hamilton Bonaduz AG, Bonaduz, Switzerland) equipped with 8 pipetting channels, a plate handler, two tools for automatic opening of special reaction tubes (FlipTubes, Hamilton Bonaduz AG, Bonaduz, Switzerland), a microtiter plate washer (405 LS, Biotek, Winooski, USA), a microtiter plate reader (Synergy HTX, Biotek, Winooski, USA) and a plate heater/shaker (Hamilton Heater Shaker, Hamilton Bonaduz AG, Bonaduz, Switzerland).

The headspace of each stirred-tank-bioreactor was rinsed with $0.1\ L\ min^{-1}$ sterile humid air. The headspace was cooled to 20 $^\circ$C to reduce evaporation during operation. The stirrer speed was constant at 3000 rpm throughout all cultivations. Parallel fed-batch processes with varying constant feeding rates were performed on a mL-scale. Substrate solution was added intermittently by the LHS with a target frequency of $6\ h^{-1}$. The precise timing of substrate addition was controlled by a scheduling algorithm and varied based on the occurrence of, for example, sampling events. The feed solution consisted of glucose ($100 - 300\ g\ L^{-1}$) and $MgSO_{4}$ ($2.5 - 7.5\ g\ L^{-1}$) with varying concentrations to allow for dosing intervals at a minimum dosage volume of 14 $\upmu$L. The pH was controlled individually at pH 6.9 by the addition of $12.5\ \% (v/v) NH_{4}OH$. To save LHS time, the pH correction was applied for all eligible reactors, i.e. when 12 out of 48 bioreactors showed a pH deviation, $12.5\ (v/v) NH_{4}OH$ was added to all 12 reactors. The frequency at which the LHS started these pH control procedures was $6\ h^{-1}$.

Isopropyl SS-D-1-thiogalactopyranoside (IPTG) with a final concentration of 0.24 to 32 $\upmu$M was added by the LHS to induce recombinant gene expression one hour after the fed-batch processes had been initiated on the mL-scale. The IPTG stock solutions were stored in closed 1.5 mL reaction tubes on the LHS workspace. During the IPTG addition procedure, the LHS opened and closed the reaction tubes automatically. IPTG concentrations were calculated based on the initial reaction volume of 10 mL. To ensure sterile operation of the LHS, the pipetting needles of the LHS were washed with an aqueous solution of 70 % (v/v) ethanol and with sterile filtered deionised water after each pipetting step.

All tasks (substrate addition, pH control, inducer addition, sampling) were initiated by a priority-based scheduler which weighed the tasks based on their real-time priority to enable optimal process control when more than one task was eligible. The detailed description of the scheduler working principle, aim and software engineering may be found in *Control of Parallelized Bioreactors I* [21]. The priorities for this application were feed > inducer addition > sampling > pH control, whereby the sampling step was further split up into sub-tasks to allow for more important tasks to be executed in between. This is one of the advantages by the scheduling architecture and allowed for a more stable feeding frequency.

### Analytical procedures

The cultivations on the L-scale were monitored online by sensor data, whereas samples on the mL-scale were generally taken every hour by the LHS, with two exceptions: (a) the first and the second sample were taken at 0.083 h and 1.25 h, respectively and (b) the last three samples were taken every two hours. Concretely, the OD data was captured by the LHS, while the pH and DO online data was captured via custom SiLA2 service implementations for the Bioreactor 48 block and PreSens sensors.

Sampling for the measurement of the optical density was conducted automatically by the LHS. Initially, samples of 150 $\upmu$L were pipetted in a microtiter plate. All samples were diluted sequentially in a second microtiter plate 1:10 and 1:100 with phosphate-buffered saline (PBS, $8\ g\ L^{-1}$ NaCl, $0.2\ g\ L^{-1}$ KCl, $1.44\ g\ L^{-1} Na_{2}HPO_{4}$, $0.24\ g\ L^{-1} KH_{2}PO_{4}$). The 1:100 diluted samples were used to measure the optical density at 600 nm ($OD_{600}$). Afterwards, both microtiter plates were washed with a microtiter plate washer (405 LS, Biotek, Winooski, USA) operated by the LHS. The sample liquids were initially aspirated and discarded followed by three dispensing and aspiration steps with 300 $\upmu$L deionised water with 0.1 % (v/v) tween (Tween 20, Amresco, Solon, USA). To estimate the cell dry weight (CDW) concentration in the stirred-tank bioreactors on a mL-scale, a linear correlation between $OD_{600}$ and CDW concentration was prepared in cultivations on a L-scale. For CDW determinations, 3 samples with 2 mL of culture broth were withdrawn during fed-batch operation and centrifuged for 5 min at 14.930 g in pre-dried and pre-weighed culture tubes. The pellet was dried for at least 24 h at 80 $^\circ$C before weighing.

### Whole-cell biotransformations

The used biotransformation procedure is adapted from Schwendenwein et al. [18]. The whole-cell biotransformations were conducted automatically at the end of the mL-scale processes in a deep-well-plate (DWP) with working volumes of 1 mL. The biotransformation consists of the conversion of 3-hydroxybenzoic acid to 3-hydroxybenzaldehyde. For detection purposes 2-amino benzamidoxime (ABAO) is added which reacts with the 3-hydroxybenzaldehyde formed in the biotransformations to 4-amino-2-(3-hydroxyphenyl)-1,2,3,4-tetrahydroquinazoline-3-oxide which can be measured photometrically at 360 nm. For all 48 sample positions, 25 $\upmu$L cell broth from the stirred-tank bioreactors on the mL-scale were mixed with 250 $\upmu$L 10 mM 3-Hydroxybenzoic acid dissolved in PBS, 500 $\upmu$L minimal medium (see section “Media”) with $10\ g\ L^{-1}$ glucose and 225 $\upmu$L PBS. For each sequential cultivation, three identical sets of calibration curves were generated. Each calibration set includes six different product concentrations. The educt solution (3-hydroxybenzoic acid) was replaced with different amounts of the product solution (12 mM 3-hydroxybenzoic aldehyde dissolved in PBS) to achieve a final product concentration in the DWP ranging from 0 to 3 mM. To have identical volumes in all calibration wells, the wells were filled up to 1 mL with PBS after the addition of cell solution and mineral medium. The biomass for all calibration samples was aspirated from the first (A1) bioreactor position of the respective experiment. All solutions required for the whole-cell biotransformations were prepared freshly for each experiment.

After preparing the initial reaction mixture for the biotransformations, the deep-well-plate was shaken at 35 $^\circ$C and 1000 rpm (Hamilton Heater Shaker, Hamilton Bonaduz AG, Switzerland). Every 1.1 hours, 50 $\upmu$L of all positions (48 sample positions and 18 calibration positions) was transferred to a microtiter plate and mixed with 50 $\upmu$L ABAO solution. The ABAO solution consisted of 10 mM ABAO dissolved in sodium acetate buffer ($3.69\ g\ L^{-1}$ sodium acetate, 3.15 % (v/v) acetic acid, 5 % (v/v) dimethyl sulfoxide, pH 4.5). Afterwards, the microtiter plate was incubated at room temperature for 45 minutes and measured photometrically at 360 nm and 600 nm in a microtiter plate reader (Synergy HTX, Biotek, Winooski, USA). The microtiter plate was washed with a microtiter plate washer (405 LS, Biotek, Winooski, USA) operated by the LHS. The sample liquids were initially aspirated and discarded followed by three dispensing and aspiration steps with 300 $\upmu$L deionised water with 0.1 % (v/v) tween (Tween 20, Amresco, Solon, USA). Finally, the remaining washing solution was aspirated and discarded and the microtiter plate was transferred by the LHS to its initial position. A total of 5 measurements including a measurement directly after biotransformation start were conducted.

### Data processing

The dataset exported from the laboratory automation platform was processed into a set of tabular DataFrame structures using pandas [22, 23]. Every unique combination of glucose feed rate and IPTG concentration was assigned a unique identifier (design_id) for identification inside the model. Likewise, every biotransformation reaction was assigned a replicate_id. The association between all experimental designs, whole-cell biotransformation reactions and relevant meta information such as assay well positions was tracked in a tabular form (“df_layout” sheet in dataset.xlsx). Reference wells of known product concentration were equally included in the dataset, hence the layout table includes a column with product concentration values where available. Measurements of absorbance at 360 nm and 600 nm, respectively, were kept in separate tables (“df_360” and “df_600” in dataset.xlsx), organized by the previously assigned replicate_id.

A generative hierarchical Bayesian model of the experimental process was built using the probabilistic programming language PyMC [24, 25]. It resembles the data generating process from experimental design via performance metrics and experimental effects to concentration trajectories and eventually predicting the resulting observations. A detailed explanation of the model will be presented in Results and Discussion. Posterior samples were obtained by MCMC sampling with the No-U-turn-Sampler (NUTS) implemented in PyMC. Diagnostics and visualizations were prepared using ArviZ and matplotlib [26–29] and probabilities were calculated from posterior samples using pyrff [30].

## Results and discussion

### Experimental design

**Fig. 1:**
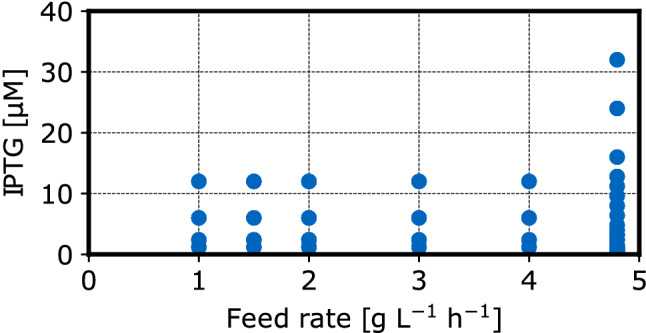
Experimental design of the experiments to identify enhanced protein production conditions for *E. coli* NoCAR: Each point depicts one unique combination of feed rate and inducer concentration that was applied during protein expression on the mL-scale. Each combination was tested in 4–8 biological replicates in total

Two variables were investigated during four parallel experiments: the glucose feed rate and the inducer concentration at the mL-scale. In total, 42 unique combinations of inducer concentration (IPTG) and feed rate (Fig. 1) were investigated with 4 to 8 biological replicates per unique combination. For controlling of the sequential batch to batch reproducibility of the mL-scale experiments, the reaction conditions at the feed rate of $2\ g\ L^{-1}h^{-1}$ were investigated twice in two sequential experiments.

### Experimental data

The conditions for the cell production phase at the L-scale and the cell transfer stayed the same throughout all four parallel experiments. After a process time of 22.75 h, a cell dry weight concentration of $13.35 \pm 1.4\ g\ L^{-1}$ was achieved with four biological replicates. This indicates that it was possible to get similar initial conditions for each of the parallel mL-scale protein expression studies.

**Fig. 2:**
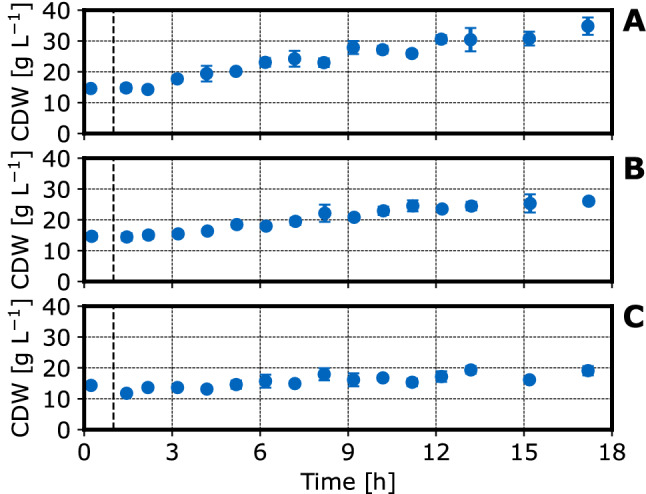
CDW concentrations measured in fed-batch operated stirred-tank bioreactors with *E. coli* NoCAR on a mL-scale: CDW concentrations were estimated based on at-line measured $OD_{600}$. The graphs depict a feed rate of **A** 4.8 $g\ L^{-1} h^{-1}$, **B** 3 $g\ L^{-1} h^{-1}$ and **C** 1 $g\ L^{-1} h^{-1}$ at inducer concentrations of **A** 0.48 $\upmu$M IPTG, **B** 6 $\upmu$M IPTG and **C** 12 $\upmu$M IPTG. The vertical dashed lines indicate the IPTG induction. Each graph shows the mean and standard deviation of 4 parallel bioreactors. (V = 10 mL, T = 30 $^\circ$C, *n* = 3000 rpm)

**Fig. 3:**
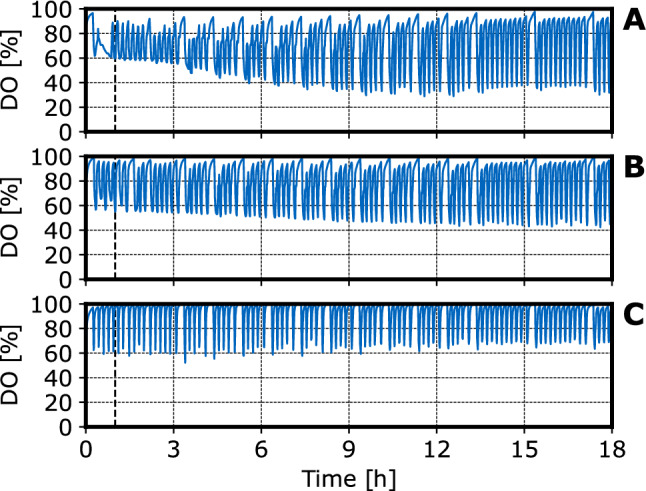
DO concentrations measured in fed-batch operated stirred-tank bioreactors with *E. coli* NoCAR on a mL-scale: The graphs depict a feed rate of **A** $4.8\ g\ L^{-1} h^{-1}$, **B** $3\ g\ L^{-1} h^{-1}$ and **C** $1\ g\ L^{-1} h^{-1}$ at inducer concentrations of **A** 0.48 $\upmu$M, **B** 6 $\upmu$M and **C** 12 $\upmu$M, respectively. The vertical dashed lines indicate the addition of IPTG. The feeding frequency was $\approx 6\ h^{-1}$. (V = 10 mL, T = 30 $^\circ$C, *n* = 3000 rpm)

**Fig. 4:**
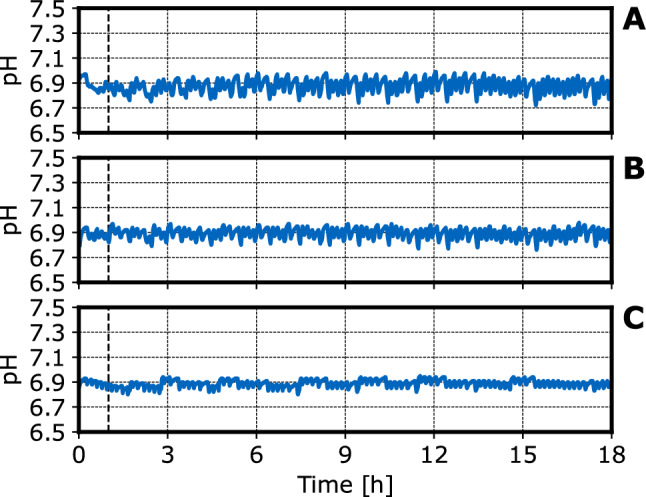
pH measured in fed-batch operated stirred-tank bioreactors with *E. coli* NoCAR on a mL-scale: The graphs depict a feed rate of **A** $4.8\ g\ L^{-1} h^{-1}$, (**B**) $3\ g\ L^{-1} h^{-1}$ and (**C**) $1\ g\ L^{-1} h^{-1}$ at inducer concentrations of (**A**) 0.48 $\upmu$M, (**B**) 6 $\upmu$M and (**C**) 12 $\upmu$M. The feeding frequency was $\approx 6\ h^{-1}$. The frequency at which the LHS added 12.5 % (v/v) $NH_{4}OH$ to adjust the pH was $\approx 6\ h^{-1}$. The vertical dashed lines indicate addition of IPTG. (V = 10 mL, T = 30 $^\circ$C, *n* = 3000 rpm)

Cell dry weight concentrations (CDW), pH and DO signals of three fed-batch processes performed on a mL-scale are shown in Figs. 2, 3 and 4.

As expected, there is a positive correlation between the applied feed rate and the cell growth. However, the biomass yields ($0.25\ g_{cells}\ g^{-1}$ glucose, $0.22\ g_{cells}\ g^{-1}$ glucose and $0.28\ g_{cells}\ g^{-1}$ glucose at feed rates of $4.8\ g\ L^{-1} h^{-1}$, $3\ g\ L^{-1} h^{-1}$ and $1\ g\ L^{-1} h^{-1}$, respectively) is lower than expected for *E. coli* growing with glucose as a carbon source [31]. This may be due to the starvation period between intermittent glucose additions with a frequency of $\approx 6\ h^{-1}$ or due to the protein production [20].

After process start, the DO rises to about 90 % air saturation (Fig. 3.). After that, the DO drops to about 40-60 % air saturation after each substrate addition with a step-time of ${\tilde{1}}$0 min followed by an increase after a few minutes due to the consumption of the glucose added intermittently. After several hours process time, the DO drop seems to be proportional to the glucose feed rate, i.e. the DO minimum after each substrate addition is approximately 40 % at a feed rate of 4.8 $g\ L^{-1} h^{-1}$ compared to 60 % at a feed rate of 1 $g\ L^{-1} h{-1}$. This is probably caused by the higher biomass density at a higher feed rate (see Fig. 2). During the first hour at a feed rate of $4.8\ g\ L^{-1} h^{-1}$, there is no increase of the DO signal indicating no limiting substrate concentrations between the substrate additions. The initially reduced metabolic activity of the recombinant *E. coli* observed at the highest feed rate may be caused by the adaption of the cells to the new cultivation temperature (37 $^\circ$C in the L-scale, 30 $^\circ$C in the mL-scale). The impact of DO fluctuations on the cell growth and protein production with *E. coli* have been studied thoroughly by Faust et al. [20]. It was found that the intermittent substrate feeding did not lead to lower final cell densities, but did reduce heterologous protein productivity for some target proteins. Further studies would be necessary to investigate whether the NoCAR expression is susceptible to intermittent substrate feeding.

The pH-setpoint for the proportional controller was pH 7.0. Due to the nature of a proportional controller, a small deviation (approx. pH 0.1) from the setpoint was observed (Fig. 4). Apart from that, the pH oscillates in a very narrow window of approximately pH 0.15 due to the intermittent pH correction by the LHS and the intermittent metabolic activity of the cells due to the intermittent feeding [32]. Overall, the pH was tightly controlled at about pH 6.9. The small pH deviations from that value will most likely be too small to have biological impact on *E. coli* growth [33, 34]. However, there might be an influence on protein expression and enzyme activity [35, 36]. Due to the intermittent dosage by the LHS, and the lower priority assigned for such tasks, those pH oscillations can not be avoided with this setup.

After 18 h of process time on the mL-scale (17 hours of protein expression) a biotransformation was prepared for each bioreactor to determine the final enzymatic activity in each bioreactor. Additionally, a calibration curve with a total of 18 positions was prepared based on the biomass from the first mL-scale bioreactor in the current experiment (A1). From the biotransformation in the Deep-Well-Plate (DWP), samples were taken every 1.1 h to measure the product concentration (360 nm) and biomass growth (600 nm) photometrically.

### Challenges in data analysis

**Fig. 5:**
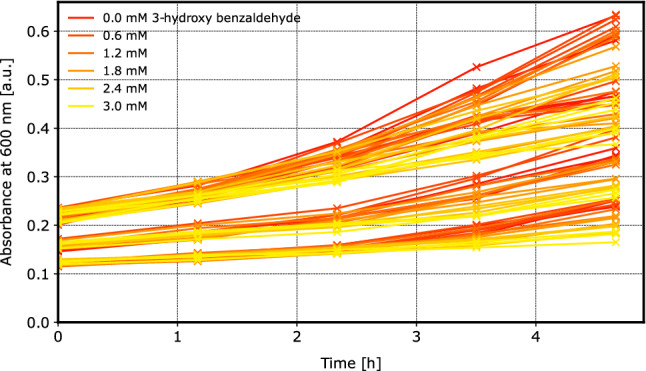
600 nm absorbance in reference wells with known 3-hydroxy benzaldehyde concentrations: Initial absorbance from biomass in the 12 reference wells varies between the experiment batches. The increase in 600 nm absorbance over time negatively correlates with the 3-hydroxy benzaldehyde concentration, indicating that formed product inhibits the growth of the whole-cell biocatalyst

A sophisticated data analysis workflow is needed to gain quantitative insight from a dataset that is not only heterogeneous due to the number of investigated conditions and observed variables, but also grows over time as more experiments are conducted. The goal is to quantify metrics that characterize the performance of the whole-cell biocatalysts produced at varying process conditions. Most importantly, these metrics must be independent of individual experimental batches to avoid drawing incorrect conclusions from “golden batch” effects. Also, the metrics and the uncertainty about them must be inter- and extrapolated towards yet untested process conditions. On the other hand, the analysis must deal with a variety of experimental effects that inevitably occur in the automated testing workflow: (a) The initial CDW concentration in all whole-cell biotransformations and reference wells of the DWP depends on the feed rate applied in the previous fed-batch processes (Fig. 5). (b) The *E. coli* cells continue to grow during the 5 h biotransformation, but the growth rate depends on the product concentration (Fig. 5). (c) The biomass contributes to absorbance at 360 nm such that product concentration can not be measured independently.

To account for all these effects simultaneously, a computational model was developed. In the following sections, various model components and results from the computational model will be introduced, starting with the calibration models needed to explain observed absorbance at 360 and 600 nm given predicted CDW and product concentrations.

### Calibration models

#### Biomass concentration

**Fig. 6:**
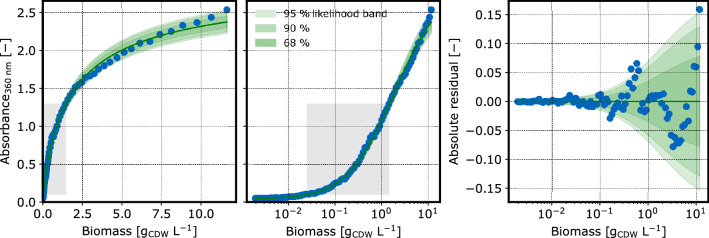
CDW calibration at 360 nm: The spread of observations (blue dots) is modeled by a calibr8.LogIndependentAsymmetricLogisticN model with scale_degree=1 to account for non-linearity (left) and heteroscedasticity (right). Green areas depict the intervals of 95 %, 90 % and 68 % probability of observations according to the model. The gray areas depict the experimentally relevant ranges

**Fig. 7:**
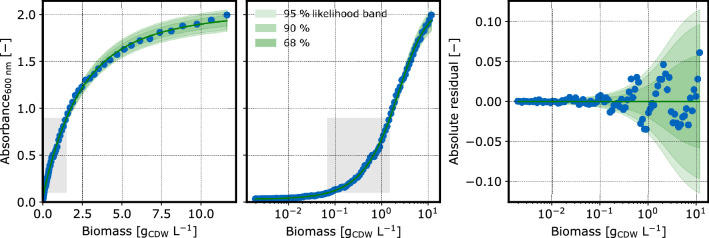
CDW calibration at 600 nm: Observations (blue dots) at 600 nm indicated lower absorbance compared to 360 nm. Like for 360 nm, the model is a calibr8.LogIndependentAsymmetricLogisticN model with scale_degree=1. The gray areas depict the experimentally relevant ranges

A separately acquired biomass calibration dataset was used to fit two models $\phi _\mathrm {cm,X,600\ nm}$ and $\phi _\mathrm {cm,X,360\ nm}$ describing the relationship between CDW concentrations and absorbance at 360 nm, and 600 nm respectively (Fig. 6,Fig. 7). The calibration at 360 nm is required to account for interference with the ABAO reaction product measurements at the same wavelength.

The models were built with the calibr8 package [37, 38] using an asymmetric logistic function of the logarithmic biomass concentration to describe the mean of normally distributed absorbance observations. Since the absorbance/CDW relationship exhibits a heteroscedastic noise, the scale parameter of the Normal distribution was modeled as linearly dependent on the mean. The models explain the observations reasonably well, even outside the experimentally relevant CDW concentration range of $0.1-0.5~g/L$.

#### Product concentration

**Fig. 8:**
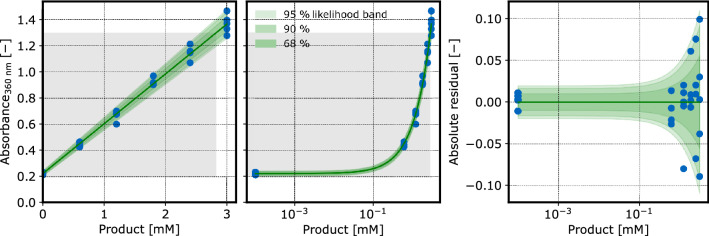
Product calibration at 360 nm: In the observed range, the absorbance at 360 nm (blue dots) followed a linear trend depending on the 3-hydroxy benzaldehyde concentration. The model was built from a calibr8.BasePolynomialModelN model with mu_degree=1 and scale_degree=1. The grey areas depict the experimentally relevant areas of the calibration

The ABAO reaction was performed to quantify 3-hydroxy benzaldehyde. The absorbance of its reaction product was measured at 360 nm in all assays. A separate calibration dataset was obtained by performing the assay procedure with reference samples of known 3-hydroxy benzaldehyde concentrations (Fig. 8). Reference samples were prepared without biomass and with different amounts of acetic acid to exclude biomass absorbance, and investigate pH robustness of the method.

A linear calibration model $\phi _\mathrm {cm,P,360\ nm}$ with heteroscedastic, normally distributed observation noise was fitted to the 360 nm measurements of product calibration samples.

All calibration model parameters were estimated by maximum likelihood using SciPy optimizers. For code and Jupyter notebooks that can be executed to reproduce this analysis we refer to the accompanying GitHub repository (https://github.com/JuBiotech/diginbio-car-paper, [39]).

### Process model

This model closely resembles the biotechnological process that generated the dataset, therefore we call it *process model* henceforth. Starting from input parameters such as specific biotransformation activity, random effects, or dependence of final 10 mL reactor CDW concentration on glucose feed rate, the process model simulates CDW and 3-hydroxy benzaldehyde concentrations in each biotransformation well across all experiments.

Table 1 summarizes the symbols, meaning, and units used in the context of the process model.

**Table 1:** Glossary of abbreviations used in the modeling context

Symbol	Unit	Meaning
BTR	n.a.	Bench-top reactor
MBR	n.a.	Macro bioreactor
DWP	n.a.	Deep-well plate
$\phi _{cm}$	n.a.	Calibration model
$\phi _{pm}$	n.a.	Process model
*X*	$g_{CDW}\ L^{-1}$	Biomass concentration
*P*	$mmol\ L^{-1}$	3-hydroxy benzaldehyde concentration
$A_{\ldots nm}$	a.u.	Absorbance at wavelength .
$\mu _X$, $\mu _P$	a.u.	Mean of absorbance readouts expected from biomass/product
$\sigma _X$, $\sigma _P$	a.u.	Standard deviation of absorbance readouts from biomass/product
${\mathcal {L}}$	–	Likelihood
$\ell$	–	Lengthscale of fluctuations in dependence on *d*
*GP*	n.a.	Gaussian process distribution
*d*	–	$log_{10}$ of the process design (feed rate, IPTG conc. or both)
$k(d, d')$	n.a.	Covariance function to obtain the kernel of a Gaussian process
*s*	$h^{-1}/(g_{CDW}\ L^{-1})$	Specific biocatalyst rate constant
*k*	$h^{-1}$	Absolute biocatalyst rate constant $\left( {\frac{{n_{{{\text{product}}}} }}{{n_{{{\text{substrate}}}} }}\;h^{{ - 1}} } \right)$

A likelihood needed for parameter inference by Markov-chain Monte Carlo (MCMC) is created from process model predictions and observed absorbance according to relationships described by the separately fitted calibration models $\phi _\mathrm {cm,X,600\ nm}$, $\phi _\mathrm {cm,X,360\ nm}$ and $\phi _\mathrm {cm,P,360\ nm}$ (1). At 600 nm this is the likelihood of the observed data given the predicted CDW concentration *X*. At 360 nm however, both biomass *X* and ABAO reaction product absorb and therefore the sum of their absorbance needs to be taken into account for the likelihood.

Note that while it is the ABAO reaction product that contributes absorbance at 360 nm we performed the ABAO assay calibration with known 3-hydroxy benzaldehyde concentrations, so the corresponding model $\phi _\mathrm {cm,P,360\ nm}$ describes 360 nm ABAO reaction product absorbance as a function of 3-hydroxy benzaldehyde concentration. For simplicity, we therefore use the symbol *P* to refer to the product of interest concentration: 3-hydroxy benzaldehyde in the biotransformation solution.

1 $$\begin{aligned} \begin{aligned} {\mathcal {L}}_{\Pi } =&\ {\mathcal {L}}_\mathrm {600\ nm}(\mathrm {A_{600\ nm}} \mid \mathrm {A_{600\ nm,obs}}) \\ \cdot&\ {\mathcal {L}}_\mathrm {360\ nm}(\mathrm {A_{360\ nm}} \mid \mathrm {A_{360\ nm,obs}}) \\ \text {where} \\ \mathrm {A_{600\ nm}} \sim&\ Normal(\mathrm {\mu _{X,600\ nm}}, \mathrm {\sigma _{X,600\ nm}}) \\ (\mathrm {\mu _{X,600\ nm}}, \mathrm {\sigma _{X,600\ nm}}) =&\ \phi _\mathrm {cm,X,600\ nm}(X) \\ \mathrm {A_{360\ nm}} \sim&Normal(\mathrm {\mu _{360\ nm}}, \mathrm {\sigma _{360\ nm}}) \\ \mathrm {\mu _{360\ nm}} =&\ \mathrm {\mu _{X,360\ nm}} + \mathrm {\mu _{P,360\ nm}} \\ \mathrm {\sigma _{360\ nm}} =&\ \sqrt{\mathrm {\sigma _{X,360\ nm}}^2 + \mathrm {\sigma _{P,360\ nm}}^2} \\ (\mathrm {\mu _{X,360\ nm}}, \mathrm {\sigma _{X,360\ nm}}) =&\ \phi _\mathrm {cm,X,360\ nm}(\mathrm {\vec {X}_{\vec {t},\vec {replicate}}}) \\ (\mathrm {\mu _{P,360\ nm}}, \mathrm {\sigma _{P,360\ nm}}) =&\ \phi _\mathrm {cm,P,360\ nm}(\mathrm {\vec {P}_{\vec {t},\vec {replicate}}}) \\ \end{aligned} \end{aligned}$$

The above observation model applies to biomass *X* and 3-hydroxy benzaldehyde concentration *P* *at every time point*, *in every replicate* of either a biotransformation reaction or reference sample (2). Reference wells of known product concentrations, but without 3-hydroxy benzoic acid, are also included in the model, albeit with the assumption that the 3-hydroxy benzaldehyde concentration remains constant over time.

2 $$\begin{aligned} \begin{aligned} \mathrm {\vec {X}_{\vec {t},\vec {replicate}}}&= \{ \mathrm {\vec {X}_{\vec {t},\vec {reference}}}, \mathrm {\vec {X}_{\vec {t},\vec {DWP}}}\} \\ \mathrm {\vec {P}_{\vec {t},\vec {replicate}}}&= \{ \mathrm {\vec {P}_{(\vec {t}),\vec {reference}}}, \mathrm {\vec {P}_{\vec {t},\vec {DWP}}}\} \\ \end{aligned} \end{aligned}$$

The process model to describe these *per replicate* and *per time point* concentrations is described in the following sections.

Since almost all process model variables are vectors or matrices, we denote dimensions by subscripts with arrows. For example, the notation $\vec {X}_{\vec {t},\vec {DWP}}$ or $\vec {P}_{\vec {t},\vec {DWP}}$ should be interpreted as 2-dimensional variables (matrices) with entries for each combination of time point and DWP well. The meanings of dimension symbols is summarized in Table 2.

**Table 2:** Dimensions in the model context

Symbol	Dimension length	Variable has elements for each of the.
$\vec {BTR}$	4	L-scale batches
$\vec {MBR}$	191	mL-scale reactor vessels
$\vec {DWP}$	191	DWP wells with active biotransformations
$\vec {replicate}$	263	DWP wells, which includes biotransformation and reference wells
$\vec {t}$	5	Time points at which observations were made
$\vec {glc}$	6	Glucose feed rates investigated
$\vec {IPTG}$	25	IPTG concentrations investigated
$\vec {design}$	42	Unique combinations of glucose feed rate & IPTG concentration

### Biomass process model

The biomass in the whole-cell biotransformation experiment is sourced from a “seed train” of cultivations in three different scales and operating modes: (a) $1\ L$ L-scale fed-batch stirred-tank bioreactor with 1 per round of experimentation. (b) $10\ mL$ mL-scale fed-batch stirred-tank bioreactor with 48 per round of experimentation. (c) $1\ mL$ biotransformation in square deep-well plate with 66 per round of experimentation.

The process model must describe biomass in each well of the biotransformation, so it can be accounted for in the 360 nm absorbance. A universally applicable activity metric, that can be interpreted independently of experimental batch effects, is desired. Therefore, the model must additionally describe biomass in a way that excludes random experimental batch effects. The first process stage at which such an experiment-independent prediction is needed, is the final biomass concentration of the 1 L batch cultivation.

Concretely, we describe the per-experiment final biomass concentration at the 1 L scale as a LogNormal-distributed variable called $\mathrm {\vec {X}_{end,\vec {BTR}}}$ with an entry for each experimental run (3). To obtain an experiment-independent prediction, we introduced $\mathrm {X_{end,batch}}$ as a *group mean prior*, also known as a *hyperprior*, around which the $\mathrm {\vec {X}_{end,\vec {BTR}}}$ is centered. The prior on $\mathrm {X_{end,batch}}$ is weakly (large $\sigma$) centered at $0.5\ g/L$, whereas actual batches should only deviate from that group mean by about $5\ \%$.

3 $$\begin{aligned} \begin{aligned} \mathrm {X_{end, batch}}&\sim LogNormal(\mu =ln(0.5), \sigma =0.5)\\ \mathrm {\vec {X}_{end, \vec {BTR}}}&\sim LogNormal(\mu =ln(\mathrm {X_{end, batch}}), \sigma =0.05) \end{aligned} \end{aligned}$$

This hierarchical structure is a common motif in Bayesian modeling since it enables a model to learn variables that are essential to the process understanding (here: $\mathrm {X_{end,batch}}$) while retaining the ability to describe the fine-grained structure of the experimental data (here: $\mathrm {\vec {X}_{end,\vec {BTR}}}$). The motif of hierarchically modeled variables was used in several places of our bioprocess model. For a thorough introduction to hierarchical modeling, we recommend [40].

The second process stage in the biomass seed train is the enzyme expression in a 10 mL scale under fed-batch conditions. Every 10 mL stirred-tank reactor was inoculated with culture broth from a 1 L reactor, hence a mapping $f_\mathrm {\vec {BTR} \rightarrow \vec {MBR}}$ yields initial biomass concentrations $\mathrm {\vec {X}_{start,\vec {MBR}}}$ by sub-indexing the $\mathrm {\vec {X}_{end,\vec {BTR}}}$ variable. The experimental design of the fed-batches comprised varying glucose feed rates and IPTG concentrations. It is plausible to assume a dependence of the final biomass concentration $\mathrm {\vec {X}_{end,\vec {MBR}}}$ on the glucose feed rate. Without any mechanistic assumptions, we lump the final biomass concentration per 1 mL-scale reactor as the product of initial biomass concentration with a positive factor $\mathrm {\vec {X}_{factor,\vec {glc}}}$ that depends on the glucose feed rate (4). Dependence of $\mathrm {\vec {X}_{factor,\vec {glc}}}$ on the glucose feed rate is modeled by a Gaussian process (4) such that our model can also interpolate and make predictions for new glucose feed rate settings.

formally, a GP is an uncountable sequence of random variables, any subset of which follows a multivariate normal (also known as “Gaussian”) distribution [41]. For understanding our model, however, it is sufficient to think of GPs as a probability distribution of *functions* that fluctuate with some lengthscale and variance. Here, this distribution over functions is used, because the model must describe $\vec {s}_{\vec {design}}$ as a function of the glucose feed rate $\vec {D}_{\vec {design}}$, but we are uncertain which function would be appropriate.

4 $$\begin{aligned} \begin{aligned} \mathrm {\vec {X}_{start,\vec {MBR}}}&= f_\mathrm {\vec {BTR} \rightarrow \vec {MBR}}(\mathrm {\vec {X}_{end,\vec {DASGIP}}}) \\ \mathrm {\vec {X}_{end,\vec {MBR}}}&= \mathrm {\vec {X}_{start,\vec {MBR}}} \cdot f_{\mathrm {\vec {glc} \rightarrow \vec {MBR}}}(\mathrm {\vec {X}_{factor,\vec {glc}}}) \\ \text {with} \\ ln(\mathrm {\vec {X}_{factor,\vec {glc}}})&= f_\mathrm {\vec {lnX}_{factor,\vec {glc}}}(log_{10}(\vec {D}_\mathrm {design,\vec {glc}})) \\ f_\mathrm {\vec {lnX}_{factor,\vec {glc}}}(d)&\sim GP(0, k(d,d')) \\ k(d,d')&= \sigma ^2 \cdot e^{-\frac{(d-d')^2}{2\ell ^2}} \\ \sigma&\sim LogNormal(ln(0.3), 0.1) \\ \ell&\sim LogNormal(ln(0.34), 0.1) \end{aligned} \end{aligned}$$

**Fig. 9:**
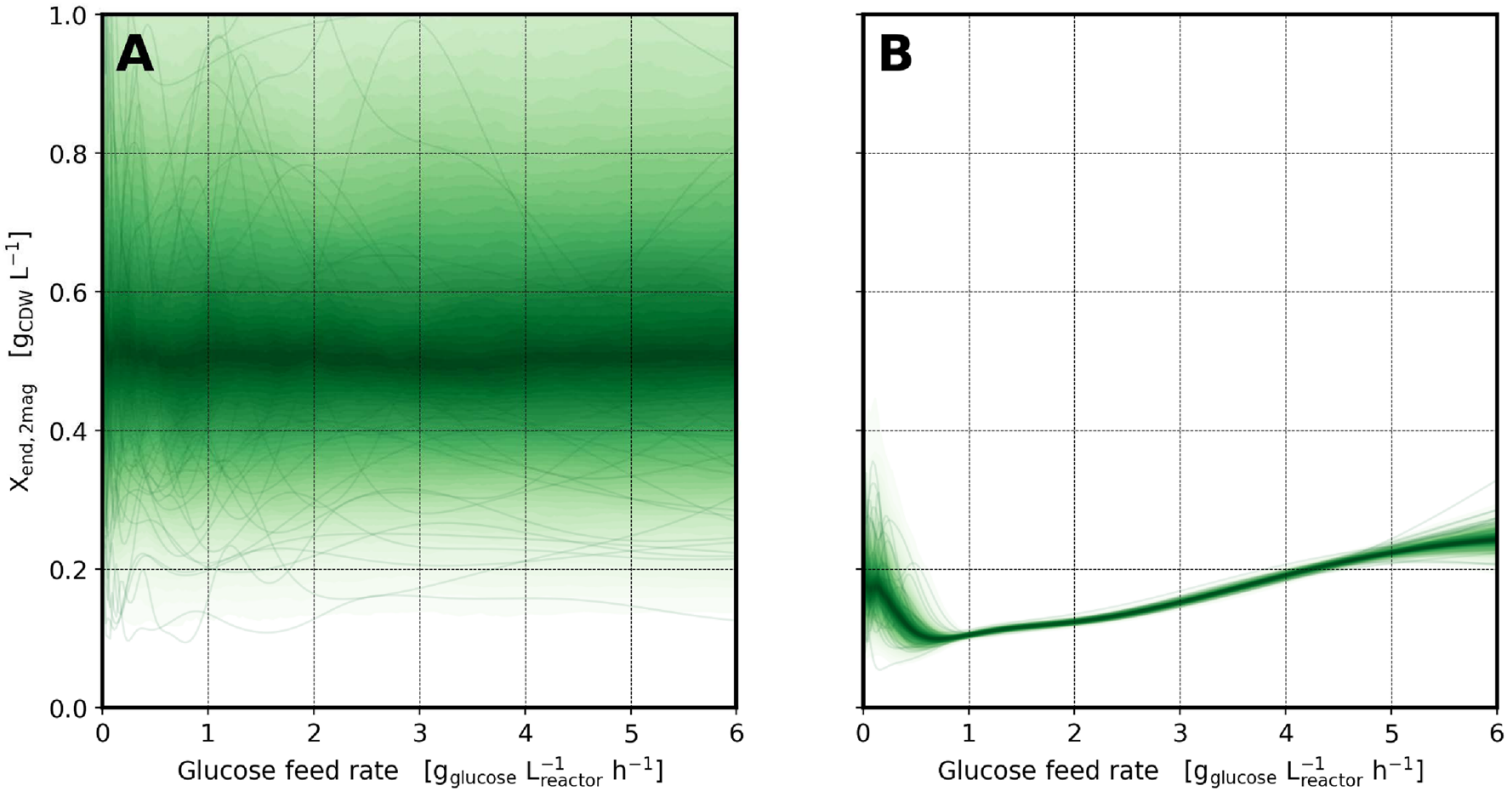
Prior and posterior of feedrate-dependent final fed-batch biomass concentration: Before observing the data (prior, left) the model predicts a broad distribution of functions (thin lines) that could describe the relationship between feed rate and final fed-batch biomass concentration. After observing the data (posterior, right), the final biomass turned out lower than expected, but the distribution of possible relationships is much narrower. Only outside the experimentally investigated range of $1.0-4.8\ g\ L^{-1}$ the uncertainty increases again

The GP was parametrized by a mean function of 0, thereby centering the prior for $\mathrm {\vec {X}_{factor,\vec {glc}}}$ around 1. For the covariance function we chose a scaling parameter $\sigma$ such that the prior variance for the factor is around $\pm 30\ \%$. The prior for $\ell$ in the exponential quadratic kernel encodes a belief that $\mathrm {\vec {X}_{factor,\vec {glc}}}$ varies smoothly on a lengthscale of around half of the (logarithmic) design space (Fig. 9).

The third and final process stage is the biotransformation. Here, the initial biomass concentration in every replicate well of the DWP $\mathrm {\vec {X}_{0,\vec {replicate}}}$ equals the final biomass concentration from a corresponding 10 mL reactor (5). The biomass concentration continued to change over the course of the biotransformation, because the solution also contained glucose as a carbon source. Inspired by the $\mu (t)$ method described in [42] we account for this biomass growth during the biotransformation with a Gaussian random walk of the discretized growth rate $\vec {\mu }_{\vec {t},\vec {replicate}}$. The result are biomass concentrations for every replicate well and measurement cycle $\mathrm {\vec {X}_{\vec {t},\vec {replicate}}}$ (5).

5 $$\begin{aligned} \begin{aligned} \mathrm {\vec {X}_{0,\vec {replicate}}}&= f_\mathrm {\vec {MBR}\rightarrow \vec {replicate}}(\mathrm {\vec {X}_{end,\vec {MBR}}}) \\ \mathrm {\vec {X}_{t \ge 1,\vec {replicate}}}&= \mathrm {\vec {X}_{0,\vec {replicate}}} \cdot e^{cumsum(\vec {\mu }_{\vec {t},\vec {replicate}} \cdot \vec {dt}_{\vec {t},\vec {replicate}})}\\ \vec {\mu }_{\vec {t},\vec {replicate}}&\sim GaussianRandomWalk(\sigma =0.1) \\ \end{aligned} \end{aligned}$$

### Biotransformation reaction process model

Next to biomass, the second important contributor to observed absorbance is the 3-hydroxy benzaldehyde concentration $\mathrm {\vec {P}_{\vec {t},\vec {replicate}}}$ that reacted with ABAO reagent. In the reference samples this concentration $\vec {P}_{(\vec {t}),\vec {reference}}$ is known and assumed to be constant. For the remaining wells it is the reaction product concentration of the biotransformation $\mathrm {\vec {P}_{\vec {t},\vec {DWP}}}$. Here we assume an initial product concentration $P_0=0$ and model the biotransformation reaction as a 1st order reaction (6) starting from a global initial benzoic acid concentration $S_0$ with a rate constant $\vec {k}_{0,\vec {DWP}}$.

6 $$\begin{aligned} \begin{aligned} \vec {P}_{\vec {t},\vec {DWP}}&= S_0 \cdot (1 - e^{-\vec {t}_\mathrm {actual,\vec {DWP}} \cdot \vec {k}_{\vec {t},\vec {DWP}}}) \\ \vec {t}_\mathrm {actual,\vec {DWP}}&= \vec {t}_\mathrm {recorded,\vec {DWP}} + t_\mathrm {delay} \\ t_\mathrm {delay}&\sim HalfNormal(\sigma = 0.1) \end{aligned} \end{aligned}$$

This well-wise rate coefficient $\vec {k}_{\vec {t},\vec {DWP}}\ [h^{-1}]$ from (6) depends on three factors. The first is the concentration of the whole-cell biocatalyst $\vec {X}_{\vec {t},\vec {DWP}}\ [g_{CDW}/L]$ as obtained from the biomass model described above. The second factor is the biocatalyst’ specific rate coefficient $\vec {s}_\mathrm {\vec {design}}\ [\frac{1}{h} / \frac{g_{CDW}}{L}]$ that depends on the experimental design of the expression phase. The third factor is a batch-wise random effect $\vec {F}_{\vec {BTR}}\ [-]$ to account for remaining experimental variability (7).

7 $$\begin{aligned} \begin{aligned} \vec {k}_{\vec {t},\vec {DWP}}&\sim LogNormal( ln(\mu _{\vec {k}_{\vec {t},\vec {DWP}}}), 0.05 ) \\ \mu _{\vec {k}_{\vec {t},\vec {DWP}}}&= \mathrm {\vec {X}_{\vec {t},\vec {DWP}}} \cdot f_1( \vec {s}_{\vec {design}} ) \cdot f_2(\vec {F}_\mathrm {\vec {BTR}}) \\ \text {with} \\ f_1&: \vec {design} \rightarrow \vec {DWP} \\ f_2&: \vec {BTR} \rightarrow \vec {DWP} \\ \end{aligned} \end{aligned}$$

For the overall bioprocess optimization study we were interested in two quantities: Design-wise specific rate coefficients $\vec {s}_{\vec {design}}$ and an experiment-independent initial rate coefficient $\vec {k}_{0,\vec {design}}$ that accounts for the biomass concentration resulting from the fed-batch expression. The $\vec {s}_{\vec {design}}$ parameter is part of the above equation and modeled by a two-dimensional Gaussian process to allow for inter- and extrapolation to new experimental designs.

$\vec {s}_{\vec {design}}$ is strictly positive, and we expect it around $0.1-0.8\ [h^{-1}]$. The model outlined in (8) achieves both properties by describing a GP for $ln(\vec {s}_{\vec {design}})$ and assigning a corresponding prior for the kernel variance $\sigma$. The prior for lengthscales $\vec {\ell }$ was centered on the half of the $log_{10}$ range (upper minus lower bound) of the design space. A similar structure was used earlier for the $\mathrm {\vec {X}_{factor,\vec {glc}}}$ variable in the upstream biomass model.

8 $$\begin{aligned} \begin{aligned} \vec {s}_{\vec {design}}&= \mu _{s_{\vec {design}}} \cdot \vec {F}_{s_{\vec {design}}} \\ \vec {\mu }_{s_{\vec {design}}}&\sim LogNormal(ln(0.75), 0.3) \\ ln(\vec {F}_{s_{\vec {design}}})&= f_\mathcal{GP}\mathcal{}( log_{10}(\vec {D}_{\vec {design}}) ) \\ f_\mathcal{GP}\mathcal{}(d)&\sim \mathcal{GP}\mathcal{}(0, k(d, d')) \\ k(d,d')&= \sigma ^2 \cdot e^{-\frac{(d-d')^2}{2\vec {\ell }^2}} \\ \sigma&\sim LogNormal(ln(0.7), 0.2) \\ \vec {\ell }&\sim LogNormal(ln(\mathrm {\vec {range}}), 0.1) \\ \vec {\mathrm {range}}&= (0.681, 2.125)^T \end{aligned} \end{aligned}$$

Finally, the initial rate coefficient metric $\vec {k}_{0,\vec {design}}\ [h^{-1}]$ is derived from model parameters that do not depend on batch/reactor/replicate-specific variables (9).

9 $$\begin{aligned} \begin{aligned} \vec {k}_{0,\vec {design}}&= \vec {s}_{\vec {design}} \cdot \mathrm {\vec {X}_{end,\vec {design}}} \\ \mathrm {\vec {X}_{end,\vec {design}}}&= \mathrm {X_{end,batch}} \cdot \mathrm {\vec {X}_{factor,\vec {glc}}} \end{aligned} \end{aligned}$$

### Modelling results

The previous three chapters outlined how trajectories of CDW concentration (Sec. 3.6) and product concentration (Sec. 3.7) were predicted and how these trajectories were fed into the three calibration models (Sec. 3.4) relating them to the observed data. Because this entire model was implemented as a symbolic computation graph, the PyMC and Aesara frameworks can auto-differentiate the likelihood (1) to obtain gradients needed for efficient MCMC sampling (Sec. 2.7).

**Fig. 10:**
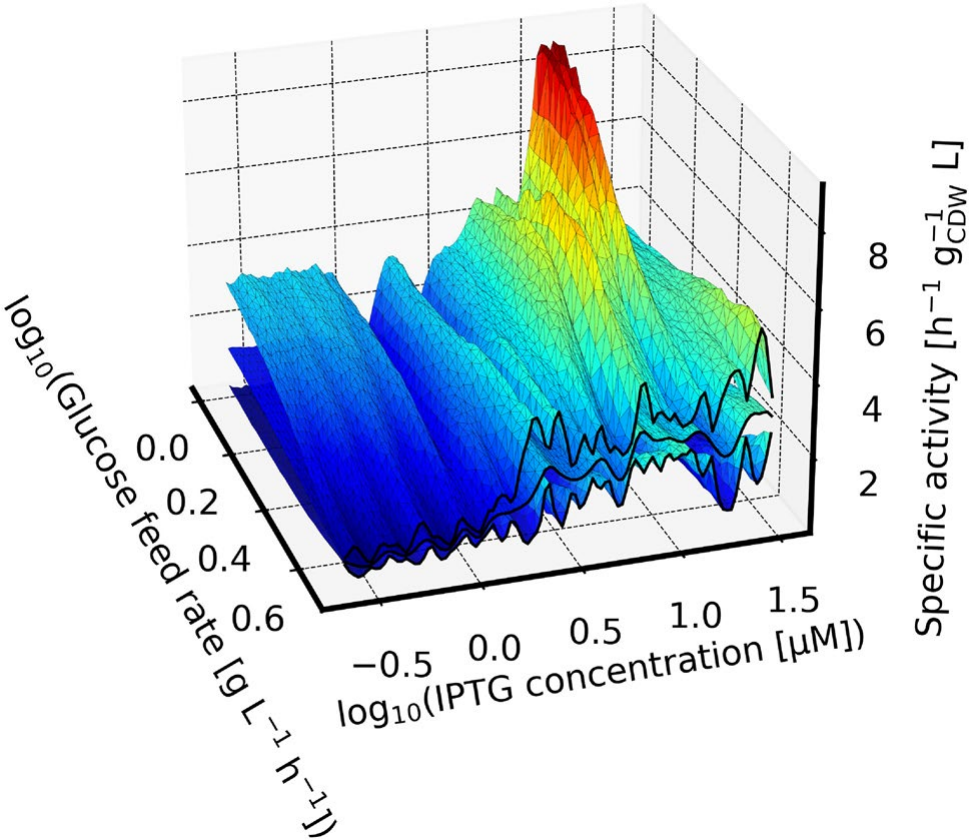
Prediction of specific activity: The surfaces show the median (center surface) and 90 % highest density interval of the posterior predictive distribution for specific activity as a function of the experimental design parameters. The highest specific activities are predicted at high IPTG concentration once in the low and once in the high feed rate regime. However, the uncertainty at lower feed rates is high which can be seen by the comparison of the rear-left with the front-left corners of the visualization. Surface color encodes the specific activity using the “Jet” colormap [43] for easier visibility

**Fig. 11:**
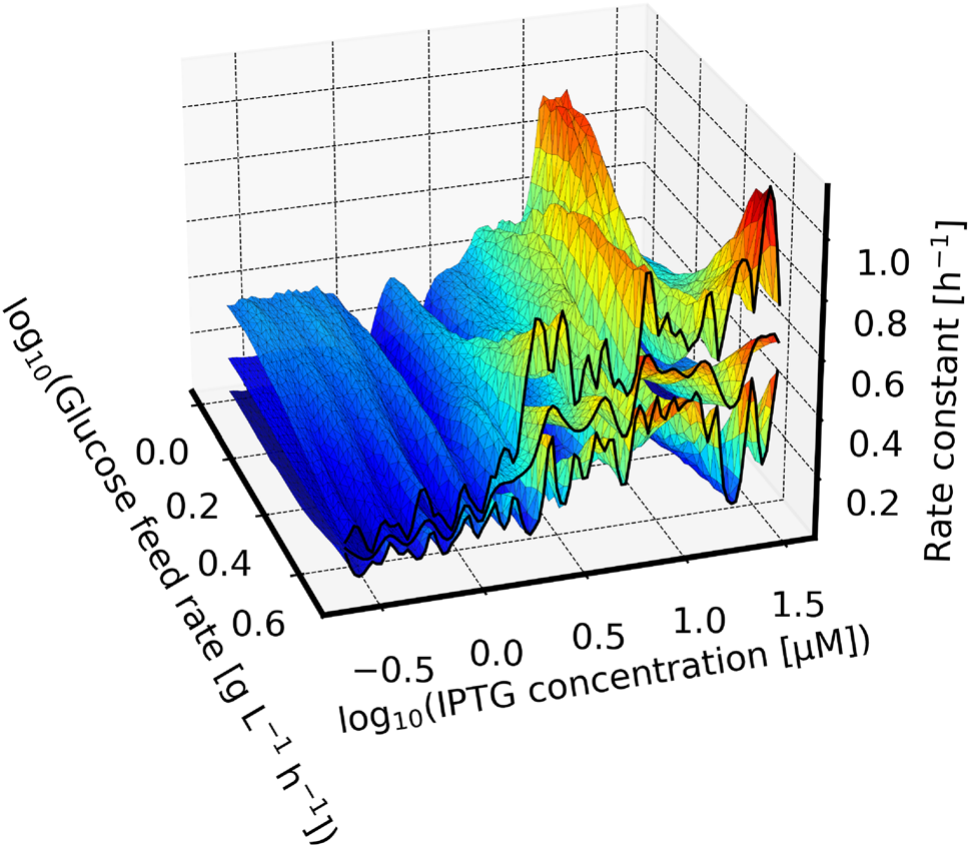
Predicted rate constants at initial biotransformation biomass concentration: The surfaces show the median (center surface) and 90 % highest density interval of the posterior predictive distribution for the rate constant to be expected from biomass suspension after the fed-batch as a function of the experimental design parameters. Surface color encodes the rate constant using the “Jet” colormap [43] for easier visibility

After MCMC sampling of the process model parameters, a variety of diagnostics, predictions and visualizations were prepared from the result. Similar to the posterior predictive distribution of the biomass/feed rate relationship (Fig. 9), the 2-dimensional Gaussian process component of the model was used to predict inter- and extrapolated specific biotransformation activity in dependence on the experimental design parameters. The visualization of the specific activity relationships posterior distribution (Fig. 10) exhibits a peak at low glucose feed rates and high IPTG concentrations. Generally, the specific activity is higher for high IPTG concentrations, but at least for high glucose feed rates where more experimental data are available (see Fig. 11) we observed the IPTG concentration to saturate at $\approx 10^{0.5} \mu M$. This observation is in line with a previous study on mCherry expression where the IPTG saturation concentration was found at $10^1 \mu M$ [12].

The highest investigated experimental design was at a feed rate of $1\ g\ L^{-1} h^{-1}$ and an inducer concentration of 12 $\upmu$M IPTG. This is more than two-fold higher than that at a feed rate of $4.8\ g\ L^{-1} h^{-1}$, yet the model predicts a comparably high specific activity at such a low feed rate. Consequently, a benefit of lower feed rate during protein expression cannot be ruled out for this protein.

**Fig. 12:**
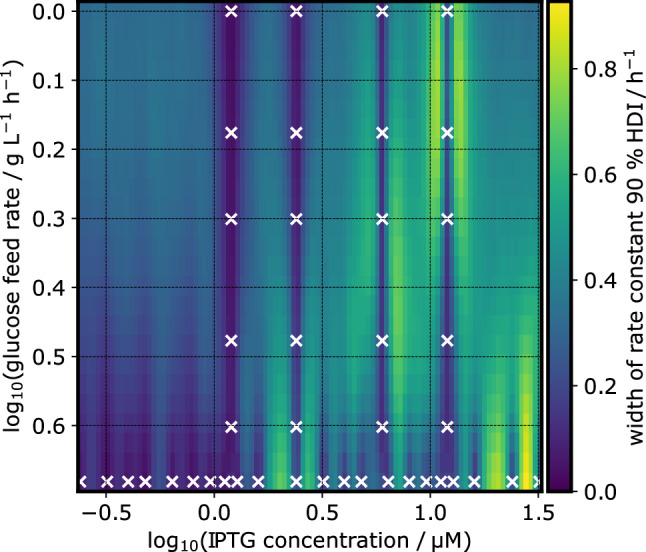
Prediction uncertainty at various process designs: The intensity of the heatmap encodes the width of the 90% highest density interval of the predicted rate constant. This measure of uncertainty is higher in regions of the parameter space where no experiments were performed. In the IPTG dimension, the model inferred a short lengthscale, leading to a quick rise of uncertainty as the distance to a data point increases (lower part). In the glucose feed rate dimension, the lengthscale is large and no oscillation of the uncertainty interval is observed (left and right parts)

The oscillatory behavior of the prediction is in line with the localization of tested experiment designs, i.e. the uncertainty rises between each investigated experimental setup. This is visualized in Fig. 12, where the width of the 90 % highest density interval—the distance between the lower and upper surface in Fig. 11 is shown as a heatmap. In future investigations, a more evenly distributed localization of tested experiment designs should help the model to make smoother predictions.

In this study, the best rate constant was predicted at a feed rate of $4.8\ g\ L^{-1} h^{-1}$ and an IPTG concentration of 27.6 $\upmu$M with $0.64\ h^{-1}$, which can be converted to an initial enzymatic activity of $1068\ U\ mL^{-1}$ (mL refers to bioreactor broth). The best tested process design was a feed rate of $1.5\ g\ L^{-1} h^{-1}$ and an IPTG concentration of 12 $\upmu$M with $0.69\ h^{-1}$, translating to a volumetric activity of $1153\ UmL^{-1}$. In a previous study, NoCAR was produced with an extremely low growth and expression temperature of 15 $^\circ$C in a batch process with complex medium in shake flasks with a final volumetric activity of approximately $26\ U\ mL^{-1}$ [15]. The low temperature was chosen to avoid the formation of inclusion bodies. Inclusion bodies are aggregates of protein that can form when a heterologous protein is expressed in *E. coli*. As inclusion bodies usually do not show enzymatic activity, they are usually not the desired product of protein expression. The risk of inclusion body formation is correlated to big proteins that are expressed fast in hosts that do not offer the proper environment for correct protein folding [11, 44, 45].

This shows that active NoCAR can be produced at a cultivation temperature of 30 $^\circ$C in defined medium. Several factors might have aided the production of active NoCAR in this study. The use of defined medium as opposed to complex medium in previous studies might have reduced inclusion body formation [11, 45]. Furthermore, the tightly controlled pH in the stirred-tank bioreactors on a mL-scale might have aided to reduce antibody formation due to pH drift [36].

**Fig. 13:**
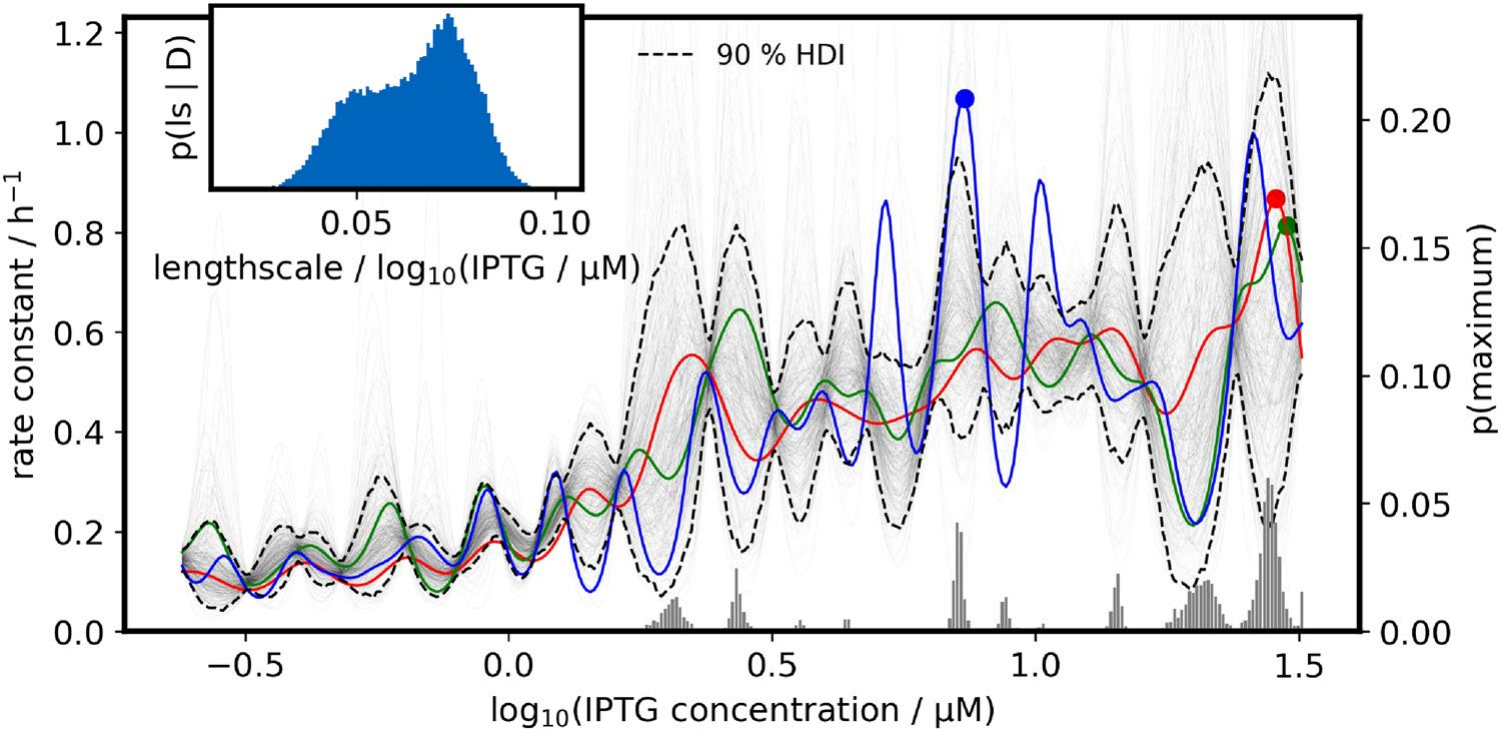
Cross-section rate constant prediction at highest glucose feed rate: Shown is the conditional posterior predictive distribution of the rate constant KPI in dependence on the IPTG concentration. Thin lines are samples from the distribution, and the red/green/blue lines highlight randomly picked examples with their maximum marked by the circle. The bar plot is the posterior probability that the maximum rate constant lies at certain IPTG concentrations, conditioned on the highest glucose feed rate. Every thin line was sampled with a different lengthscale from the posterior distribution shown in the inset plot

The oscillations in the two-dimensional uncertainty shown in Fig. 11 and Fig. 12 are the result of the underlying Gaussian process model that describes possible functions of $s_\text {design}$ dependence on the two process design parameters. Fig. 13 shows posterior predictive samples of that Gaussian process model, conditioned on the highest glucose feed rate. In essence, Fig. 13 is a more detailed cross-section that is marked by black lines in Fig. 11. Note that the GP samples are drawn with different lengthscales, hence some may fluctuate more smoothly than others. Again, a more evenly spread localization of experimentally tested process designs should help to smoothen the prediction by providing more information about the spatial dependence, at the expense of higher uncertainty at individual process designs.

Our model found lower feed rates to be possibly beneficial for specific activity (Fig. 10), even after taking the resulting biomass concentration into account (rate constant, Fig. 11). At the same time, the model is still undecided about the lengthscale of IPTG dependency (Fig. 13, inset plot). At short lengthscale the functions drawn from the Gaussian process are rougher (fluctuate faster) which widens the uncertainty faster as the distance to a tested process design increases. With long lengthscale on the other hand the functions are smoother, and fluctuate less between the designs. The Gaussian process in Fig. 11 makes an uncertain extrapolation of this trend towards lower feed rates where the density of observations was much lower. Counterintuitively this leads to a vague prediction that the optimal process design could be at lower feed rates and moderately high IPTG concentration.

**Fig. 14:**
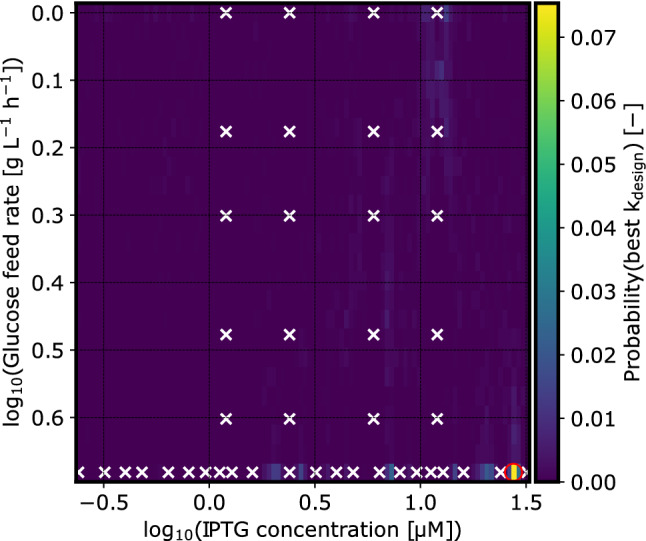
Probability landscape of the rate constant optimum within the investigated design space: For each process design in a 50x50 grid of process parameters the probabilistic prediction of the rate constant metric was translated into a probability. The intensity of the pixel indicates the probability that this particular design is the best among all 2500 combinations. Most probability is concentrated in a region of low glucose feed rates combined with high IPTG concentrations. The red circle marks the combination that was predicted to be optimal with the highest probability

The probability map (Fig. 14) is a more direct visualization of this prediction. The overlaid coordinates of experimentally tested process parameters show that this part of the parameter spaces has not been extensively investigated yet.

## Conclusion

The automated cascade of stirred-tank bioreactors enabled screening of 42 different combinations of inducer concentration and feed rate during protein expression of *E. coli* NoCAR in a scalable bioreactor setup. A total of 192 bioreactor runs were performed during four weeks, showing the high productivity of miniaturized, automated and digitized parallel bioreactors. The new automated biotransformation procedure at the end of each process enabled the investigation of the enzymatic activity of each expression condition without manual intervention.

Due to the sophisticated mechanistic modelling based on Bayesian statistics, the enzymatic activity was estimated without the need of cell separation. This makes automation much simpler, because cell separation with automated liquid handling systems is costly and requires a lot of space in the working area of the robot. Furthermore, the probabilistic analysis opens the door for iterative Bayesian optimization that can further accelerate the identification of the optimal process conditions, while reducing the needed experimental effort.

At the optimal investigated expression conditions, an activity of $1153\ U\ mL^{-1}$ was estimated with a $90\ \%$ credible interval of $[992, 1321]\ U\ mL^{-1}$. Taking the uncertainty into account, this is about 38 to 50-fold higher than the highest published data for the enzyme under study. It would be interesting for further studies to investigate parameter combinations that are predicted to be beneficial by the model.

The combination of cultivation at L- and mL-scale is a rather generic experimental strategy to produce biomass for whole-cell biocatalysis under varying expression conditions. Similarly, the technique to reproduce the structure of an experimental process in a probabilistic model can be transferred to other bioprocess characterization workflows. For example, one could easily adopt the workflow to optimize other expression conditions such as pH or medium composition, by modifying the Gaussian process model for the $s_{\vec {design}}$ variable to take other process design parameters as its input. Likewise, the biotransformation reaction of interest could be replaced by another first-order reaction. Also higher order reaction kinetics could be incorporated by replacing the 1st order reaction with, for example an ODE model, that describes Michaelis–Menten, Hill- or cascade enzyme kinetics. We conclude that conducting biotransformations at higher throughput and analyzing the data with Bayesian modeling is a versatile and promising approach to accelerate the development of biocatalytic processes.
